# Isolation and Identification of Black Oil-Degrading Bacteria From Lubricant-Contaminated Soil in Northern Baghdad, Iraq

**DOI:** 10.1155/tswj/4009105

**Published:** 2025-04-14

**Authors:** Zeena Ghazi Faisal, Mohannad Mohammed Jameel, Othman Abbas Abdullah

**Affiliations:** Department of Biology, College of Education, Al-Iraqia University, Baghdad, Iraq

**Keywords:** *Bacillus cereus*, bioremediation, black oil, *Pseudomonas aeruginosa*

## Abstract

Black oil is a refined petroleum product that poses a significant environmental risk. It contains complex multihydrocarbons that decompose slowly, so black oil remains in the environment for a long time, causing various toxic effects. This study isolates and identifies an indigenous bacterium from soil samples contaminated with waste lubricating oil and evaluates its potential for degrading black crude oil. Twelve species of black oil-degrading bacteria were isolated from six soil samples of automobile and motorcycle workshops in the Al-Tarmiyah District of Baghdad, Iraq. Isolated bacteria were identified based on morphological and VITEK 2 system as *Pseudomonas aeruginosa*, *Bacillus cereus*, *Burkholderia cepacia*, *Pseudomonas fluorescens*, *Acinetobacter lwoffii*, *Acinetobacter radioresistens*, *Stenotrophomonas maltophilia*, and *Streptococcus parasanguinis*. Among these, based on the measurement of optical density and chromatogram analysis, *B. cereus* exhibited the maximum efficiency in degrading black oil, followed by *P. aeruginosa*. Therefore, these indigenous bacteria have the potential to be used in black oil removal from contaminated sites and the expansion of bioremediation approaches.

## 1. Introduction

Environmental pollution generated from petroleum product spills has become a worldwide ecological and economic disaster [1]. These spills have a devastating impact on the soil, surface and groundwater, in addition to changing the microbial population at the contaminated sites [2]. The complicated structure of petroleum can trigger multiple toxic effects on both plants and animals in the soil through contact toxicity, as well as by lowering oxygen concentrations and promoting anaerobes, which harm plant roots [3].

Black oil (mazut) is an end product of crude oil distillation and regarded as one of the most hazardous refined oil products for ecosystems and is a common pollutant in Iraq [4]. It contains resins, solid paraffin, asphalten, sulphuric compounds, long chain and cyclic alkanes, polycyclic aromatics with lateral chains, heavy metals, and unsaturated compounds, making it a highly viscous product [5]. Black oil is slowly degraded; therefore, it can survive in the environment for a long time, causing various toxic effects through the possibility of some components accumulating within susceptible organisms and being transferred to other levels in the food chain [6].

Conventional remediation processes are inappropriate due to their high application costs and the hazardous intermediates they produce. Microbial bioremediation, using bacteria, yeasts, or fungi, is a better option for restoring sites contaminated with crude oil. It is a simple, effective, economical, and ecofriendly technique compared to physical and chemical methods [6, 7]. It is beneficial to isolate microorganisms from their native habitats that meet their needs. Microorganisms are adapted to thrive in these environments, resulting in high microbial performance in pollutant utilization [8]. Several studies have shown that various genera of bacteria, including *Pseudomonas* spp., *Bacillus* spp., *Acinetobacter* spp., *Flavobacterium* spp., *Corynebacterium* spp., *Providencia* spp., *Brevibacterium* spp., and *Arthrobacter* spp., are common hydrocarbon degraders [9–11]. *Pseudomonas* and *Bacillus* species are recognized as proficient degraders of hydrocarbons by utilizing them as sole sources of carbon and energy [2, 12–15]. These bacteria evolved their enzymatic systems, enabling them to utilize such compounds as a carbon and energy source and eventually convert them into nontoxic and ecofriendly products [4, 11].

Some studies have focused on the bioremediation of black oil. Khorasani and his colleagues Khorasani et al. [6] isolated the indigenous *Enterobacter cloacae* BBRC 10061 strain from oil-contaminated soil in Mashhad, Iran. This bacterium is considered the first strain capable of biodegrading black oil. They indicated that the gradual addition of black oil to the medium could help the *E. cloacae* BBRC 10061 gradually adapt to the heavy hydrocarbon, which increases the degradation rate and biomass.

In the present work, we focused on isolating and identifying local bacteria from soil samples contaminated with lubricants, with the goal of recognizing strains capable of utilizing and effectively degrading black oil as a carbon and energy source.

## 2. Material and Methods

### 2.1. Black Oil Sample

In a dark, tightly sealed bottle, a sample of black oil was collected from the Al Doura refinery located southeast of Baghdad, Iraq. It was transferred to the laboratory and kept in a cold and dark place until use.

### 2.2. Collection of Soil Samples

**Figure 1:**
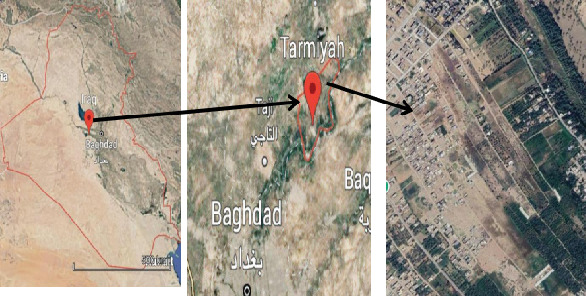
Location map of the study area.

Samples of lubricant contaminated soil were collected from the topsoil (5–15 cm) of six automobile and motorcycle workshops situated in the Al-Tarmiyah District in northern Baghdad, Iraq (Table 1 and Figure 1). The samples were stored in sterile, tightly closed, labeled polyethylene bags, transferred to the laboratory aseptically, and refrigerated at 4°C until use.

### 2.3. Isolation and Purification of Black Oil-Degrading Bacteria

To isolate black oil-degrading bacteria from the soil samples, a selective enrichment method using mineral salt medium (MSM), was used. MSM contained (grams/liter): KH2PO4 (1.0), K2HPO4 (1.0), NaCl (1.0), CaCl2 (0.05), (NH4)2 SO4 (1.0), MgSO4.7H2O (0.5), FeCl3 (0.002), and yeast extract (0.1) [14]. Enrichment was performed using 1 g of soil in 250 mL Erlenmeyer flasks containing 100 mL of sterilized MSM with 2% black oil as the sole carbon source. The flasks were incubated in a shaker incubator (150 rpm) at 30°C. After 7 days of incubation, 1 mL of the first culture was transmitted into 100 mL of fresh MSM with 2% black oil and incubated under the same conditions again. This procedure was repeated three times to mitigate the microbial load [14, 15]. After three cycles of enrichment, serial dilutions of each culture were performed, starting from 10^−1^ to 10^−7^, and then 0.1 mL from each dilution was spread onto nutrient agar plates that were incubated at 30°C for 24 h. Depending on the morphological differences, bacterial colonies were selected and purified on nutrient agar plates by the streaking method. This process was repeated until obtaining pure colonies that could be stored as aliquot slants at 4°C and subculturing at 2 weeks intervals. In nutrient agar media, the phenotypic properties of growing colonies were identified by their color, shape, size, and texture. On the other hand, microscopic examination of bacterial isolates was identified by gram stain reaction.

### 2.4. Identification of Bacteria

The VITEK 2 compact device was used for the accurate identification of bacterial isolates. This system depends on a series of biochemical tests done together, using VITEK2 gram-negative (GN), VITEK2 gram-positive (GP), and VITEK2 BCL cards. Firstly, GN bacteria were grown overnight on a blood agar medium, while GN bacteria were grown on a Macconkey agar medium. A 0.5-mL of bacterial suspension was diluted to 1.5 × 10^7^ CFU/mL in 0.45% NaCl saline. Cards were automatically filled, sealed, and loaded into the VITEK 2 instrument for incubation and reading. To identify organisms, the VITEK 2 software compares the obtained profiles with Taxa profiles in the database and assigns a percentage to each test. The qualitative level of identification was assigned depending on the calculation of numerical probability [12].

### 2.5. Estimating the Ability of Bacterial Isolates to Degrade Black Oil

An inoculum (3% v/v) of overnight bacterial culture with an optical density of 0.1 (OD_600_) was inoculated into 100 mL of MSM enriched with 2% (*v*/*v*) black oil as substrate and kept at 30°C within a shaker incubator set at 150 rpm. Subsequently, bacterial growth was assessed on the 7^th^, 14^th^, and 21^st^ days of incubation using a UV/Vis spectrophotometer at 600 nm (OD_600_) compared to MSM as a blank [16]. The experiment was performed in triplicate. The isolates with a high growth capability were selected as the efficient black oil-degrading bacteria.

### 2.6. Gas Chromatography–Mass Spectrometric (GC-MS) Analysis

From cultures of efficient degrading bacteria grown for 14 days, the residual black oil was collected by chloroform (3 sample:1 chloroform *v*/*v*) using a 500-mL separating funnel with continuous shaking. MSM with black oil was used as a control. The contents are allowed to settle, forming two layers: an aqueous layer and an organic layer containing the residual black oil. The organic phase was collected in a glass Petri dish and dried at 40°C–45°C. The precipitate was analyzed by GC-MS. The instrument contains a ZB-5MS capillary column and uses helium (He) as a carrier gas with a flow rate of 2 mL/min. The initial temperature of the column was held at 80°C for 3 min and then raised to 280°C for 10 min at a rate of 8°C/min. Before the analysis of samples by GC-MS, 0.1 g of samples were dissolved in chloroform at a concentration of 100 *μ*g/mL, and then 1 *μ*L of sample was used with a split ratio of 10:1.

### 2.7. Data Analysis

The computerized database structure was used to perform statistical analyses and report on the obtained data. For more than two groups, one-way analysis of variance (ANOVA) was performed, and *p* < 0.05 was the acceptable statistical significance.

## 3. Results and Discussion

### 3.1. Isolation and Purification of Black Oil-Degrading Bacteria

Twelve different black oil-degrading bacteria were isolated using a selective enrichment method (Table 2). The process was carried out in multiple cycles to make sure that isolates obtained at the end of enrichment cycles were capable of utilizing black oil rather than just tolerating it. Isolated bacteria are considered the active degraders of black oil. This demonstrates the ability to collect numerous bacterial isolates capable of living and utilizing black oil from soil samples that have been contaminated with petroleum or their derivatives. However, the number of bacterial isolates found at each site was different. This may be related to the diversity of bacteria capable of decomposing hydrocarbons and their derivatives, and the longer contamination time could lead to a greater number of microorganisms [17]. The results showed that GN bacteria are the dominant hydrocarbon degraders; of these, 9 (75%) belonged to the GN group, and 3 (25%) were GN.

These results agree with previous studies, which indicate the possibility of isolating various types of bacteria that possess a good ability to utilize crude oil and hydrocarbon residues from petroleum-contaminated soil [9, 12–14]. Moreover, GN bacteria were dominant because they possess efficient enzymatic systems responsible for degrading complex compounds [12, 14]. Additionally, the outer membrane of GN bacteria is oleophilic, which allows them to attach more hydrocarbons to be oxidized as a source of carbon used in growth and biomass production [18].

### 3.2. Identification of Black Oil-Degrading Bacteria

According to the results of the VITEK 2 technique, four isolates were identified as *Pseudomonas aeruginosa*, two isolates as *Bacillus cereus*, and one isolate belonged to each of *Burkholderia cepacia, Pseudomonas fluorescens*, *Acinetobacter lwoffii*, *Acinetobacter radioresistens, Stenotrophomonas maltophilia*, and *Streptococcus parasanguinis* (Table 3). The VITEK 2 system can be considered a handy tool used to confirm the results of conventional identification and to diagnose nonidentified bacterial isolates, due to its simplicity, rapid identification, taxonomically updated databases, and high level of automation [3].

Results revealed that *Pseudomonas aeruginosa* is the most common bacteria in contaminated soils, followed by *Bacillus cereus*. All other bacterial species, *Burkholderia cepacia, Pseudomonas fluorescens, Acinetobacter lwoffii*, *Acinetobacter radioresistens, Stenotrophomonas maltophilia*, and *Streptococcus parasanguinis,* play an essential role in the environment and are effective in decomposing hydrocarbons and their derivatives [19]. The special abilities of these bacterial species have been attributed to their diverse metabolic capacity to decompose hydrocarbons and recycle metals in nature [18]. Additionally, many bacterial species can generate surface-active compounds with diverse chemical structures, including glycolipids, lipopeptides, fatty acids, polysaccharide–protein complexes, and neutral lipids. These molecules improve the bioavailability of hydrocarbon contaminants and their derivatives and minimize interfacial and surface interaction, thereby facilitating an efficient emulsification process [20].

### 3.3. Estimating the Ability of Bacterial Isolates to Degrade Black Oil

In this study, the ability of bacteria to utilize black oil was evaluated by measuring the optical density (OD_600_) of bacteria grown on MSM containing 2% black oil as the sole source of carbon and energy after 7, 14, and 21 days of incubation. Bacterial growth is an indicator of the capacity to degrade black oil. The variation in OD was associated with the density of bacterial growth, as well as the breakdown of the molecular structure of black oil due to its utilization by bacteria as a carbon and energy source.

Through the first 3 days of incubation (lag phase), there was no discernible change in turbidity. This period is responsible for the bacteria adapting to their new environment in preparation for the next stage (the experiential phase). Significant growth was observed on the seventh day of incubation, with turbidity (OD_600_) ranging between 0.38 ± 0.04 and 1.09 ± 0.01, indicating significant differences (*p* < 0.05) in their ability to utilize black oil (Table 4). Bacterial growth gradually increased, reaching its maximum after 14 days of incubation.

Among the bacterial strains, *B. cereus* (S6 and S12) showed a higher OD value of 1.21 ± 0.01 and 1.19 ± 0.03, respectively. *Bacillus* species can produce spores, enabling them to survive in various environmental niches, including hydrocarbon-polluted soils. They are also known for their ability to produce biosurfactants/bioemulsifiers that contribute significantly to bioremediation efforts and natural recycling of nutrients in ecosystems [21, 22]. *P. aeruginosa* (S2, S5, S9, and S11) showed a variation in OD value ranging between 0.74 ± 0.04 for S5 to 1.12 ± 0.01 for S11. This variation is mainly due to its diverse metabolic capacity to decompose hydrocarbons [18]. *Pseudomonas* species are widely distributed in nature and known for utilizing a wide range of organic substrates for growth. These bacteria can thrive in crude oil rich in organic compounds and produce surface-active compounds that degrade crude oil [20]. *A. lwoffii*, *A. radioresistens*, *B. cepacia*, *S. parasanguinis*, *P. fluorescens*, and *S. maltophilia* demonstrate the ability to utilize black oil as a carbon and energy source, with OD values of 0.70 ± 0.04, 0.89 ± 0.02, 1.10 ± 0.02, 0.96 ± 0.03, 1.11 ± 0.02, and 1.09 ± 0.02, respectively. These species are known to produce biosurfactants that enhance emulsification and improve the bioavailability of hydrocarbons [1, 14, 18, 22]. The high enzymatic capacity of microbial communities enables the degradation of complex hydrocarbons, including aliphatics and polyaromatics.

After 21 days of incubation, all bacterial isolates showed a decline in cell growth, indicated by a decrease in optical density (Table 4). This decline is attributed to bacterial death resulting from the depletion of carbon sources. According to Xu et al. [23], petroleum hydrocarbons have a toxic effect on living organisms, and high hydrocarbon concentrations can inhibit bacterial growth and even lead to death.

### 3.4. GC-MS Analysis

**Figure 2:**
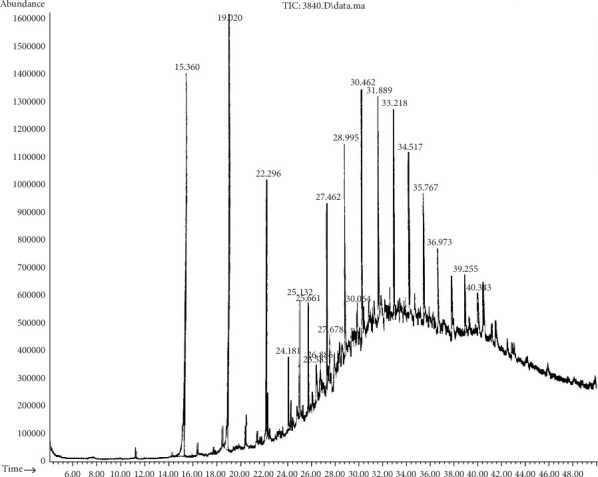
Chromatogram analysis of black oil (the control group). The different peaks showing the differences in the number of carbon atoms between the compounds of black oil.

By GC-MS analysis, the most efficient degrading bacteria (*B. cereus* (S6) and *P. aeruginosa* (S11)) were selected to determine the residual black oil and compare it to the control.  Table 5 shows the complex combination of black oil (the control group), which consists of 25 compounds of aromatic and aliphatic substances. Moreover, different peaks in Figure 2 represent the differences between compounds of black oil due to the variation in the number of carbon atoms.

**Figure 3:**
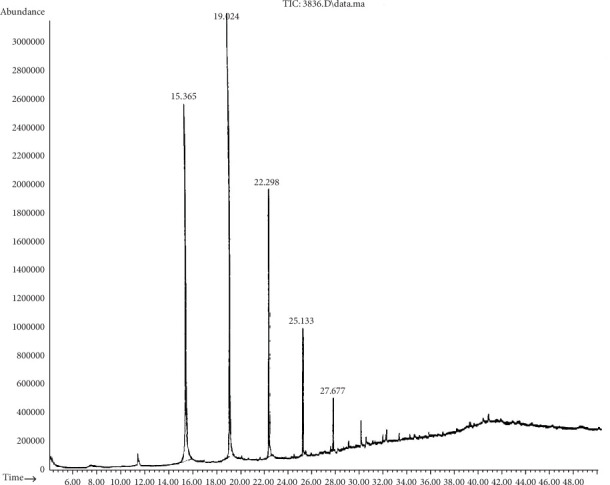
Chromatogram analysis of residual black oil degraded by *B. cereus* (S6). A reduction in the number of peaks reflects a reduction in black oil components.

**Figure 4:**
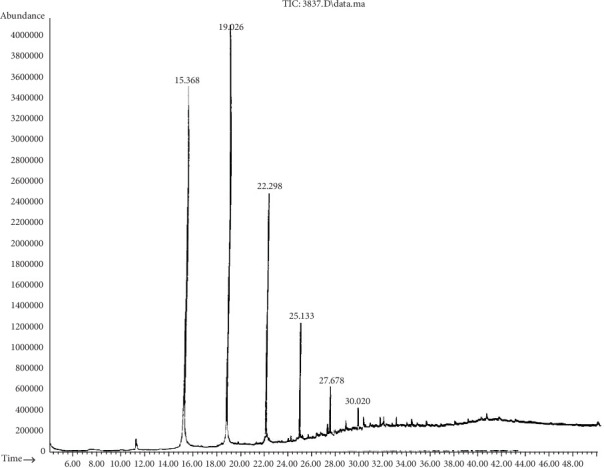
Chromatogram analysis of residual black oil degraded by *P. aeruginosa* (S11). A reduction in the number of peaks reflects a reduction in black oil components.

Chromatogram analysis of *B. cereus* (S6) and *P. aeruginosa* (S11) cultures shows a reduction in black oil compounds compared to the control (Tables 6 and 7). Figures 3 and 4 show the disappearance of several peaks compared to a control sample as the number of chemical components decreased. The appearance of new peaks indicates the formation of new intermediates that formed during the degradation of long and complex carbon chains. It is noteworthy that any GC reduction between the control and inoculated samples is referred to as a biodegradation process because any loss caused by nonbiological factors would have the same impact on the control sample as on the inoculated samples. This indicated that *B. cereus* and *P. aeruginosa* are significant black oil decomposers.

Over millions of years, bacteria evolved catalytic enzymes for the hydrocarbons degradation, making them adaptable to polluted environments. On the other hand, bacteria possess a flexible metabolism and several genes that enable them to exploit various carbon sources for energy [13]. Furthermore, many species of bacteria are known to produce surface-active compounds that can emulsify hydrocarbons, reduce surface tension, and increase the accessibility of the decomposers to oil grains [1, 14, 18, 22]. *Bacillus cereus* was reported to produce surfactin [24], whereas *P. aeruginosa* produces rhamnolipids [14].

## 4. Conclusions

The current study demonstrates that waste lubricate–contaminated soil is a good source for isolating black oil-degrading bacteria, which may be employed in bioremediation. Moreover, GN bacteria predominate. *Pseudomonas* is the most common indigenous bacteria, followed by the genera *Bacillus* and *Acinetobacter*. After 14 days of incubation in MSM with 2% black oil, *Bacillus cereus* exhibited maximum efficiency in degrading black oil, followed by *P. aeruginosa*. Consequently, these bacteria can be used in the biodegradation of hydrocarbon-contaminated soils.

## Figures and Tables

**Table 1:** The GPS coordinate of the sampling sites.

**Site no.**	**GPS coordinate**
Site 1	33°39⁣′58⁣^″^ N 44°23⁣′37⁣^″^ E
Site 2	33°39⁣′55⁣^″^ N 44°23⁣′36⁣^″^ E
Site 3	33°39⁣′55⁣^″^ N 44°22⁣′59⁣^″^ E
Site 4	33°39⁣′58⁣^″^ N 44°23⁣′00⁣^″^ E
Site 5	33°39⁣′58⁣^″^ N 44°23⁣′15⁣^″^ E
Site 6	33°39⁣′59⁣^″^ N 44°23⁣′55⁣^″^ E

**Table 2:** Morphological and microscopic examination of bacteria isolated from the topsoil contaminated with a waste lubricant that degrades black oil.

**No.**	**Isolate symbol**	**Colony color**	**Colony shape**	**Colony size**	**Colony texture**	**Gram stain**	**Cell shape**
1	S 1	White	Round, convex	Small	Mucoid	—	Rod
2	S2	Brown to green	Irregular, convex	Large	Mucoid	—	Rod
3	S3	Grey to green	Irregular, convex	Very small	Mucoid	—	Rod
4	S4	Brown to green	Round, flat	Small	Mucoid	+	Cocci
5	S5	Brown to green	Irregular, convex	Large	Mucoid	—	Rod
6	S6	Gray white	Flat, wavy edges	Large, opaque	Dry	+	Rod
7	S7	Pale yellow	Round, convex	Small	Mucoid	—	Rod
8	S8	White to yellow	Round, convex	Large	Mucoid	—	Rod
9	S9	Brown to green	Irregular, convex	Large	Mucoid	—	Rod
10	S10	Pale yellow	Circular, convex	Small	Mucoid	—	Diplococcoid rods
11	S11	Brown to green	Irregular, convex	Large	Mucoid	—	Rod
12	S12	Gray white	Flat, wavy edges	Large, opaque	Dry	+	Rod

*Note:* **+** = gram positive; − = gram negative.

**Table 3:** The identification and percentage probability of black oil-degrading bacteria using the VITEK 2 technique.

**No.**	**Isolate symbol**	**VITEK 2 identification**	**Probability (%)**
1	S 1	*Acinetobacter lwoffii*	96
2	S2	*Pseudomonas aeruginosa*	98
3	S3	*Burkholderia cepacia*	99
4	S4	*Streptococcus parasanguinis*	95
5	S5	*Pseudomonas aeruginosa*	97
6	S6	*Bacillus cereus*	99
7	S7	*Pseudomonas fluorescens*	90
8	S8	*Stenotrophomonas maltophilia*	92
9	S9	*Pseudomonas aeruginosa*	91
10	S10	*Acinetobacter radioresistens*	92
11	S11	*Pseudomonas aeruginosa*	97
12	S12	*Bacillus cereus*	96

**Table 4:** The optical density (OD_600_) of bacterial strains grown on MSM containing 2% black oil as the sole source of carbon and energy after 7, 14, and 21 days of incubation. S6 and S11 are the most efficient black oil-degraders.

**No.**	**Symbol**	**Bacterial strain**	**The optical density (OD**_**600**_ **) after**		
			**7 days**	**14 days**	**21 days**
1	S 1	*Acinetobacter lwoffii*	0.38 ± 0.04	0.70 ± 0.04	0.21 ± 0.04
2	S2	*Pseudomonas aeruginosa*	1.03 ± 0.01	1.07 ± 0.01	0.72 ± 0.04
3	S3	*Burkholderia cepacia*	0.91 ± 0.07	1.10 ± 0.02	0.76 ± 0.03
4	S4	*Streptococcus parasanguinis*	0.81 ± 0.04	0.96 ± 0.03	0.41 ± 0.02
5	S5	*Pseudomonas aeruginosa*	0.59 ± 0.03	0.74 ± 0.04	0.35 ± 0.04
6	**S6**	***Bacillus cereus***	0.98 ± 0.06	1.21 ± 0.01	0.55 ± 0.04
7	S7	*Pseudomonas fluorescens*	1.08 ± 0.07	1.11 ± 0.02	0.75 ± 0.03
8	S8	*Stenotrophomonas maltophilia*	0.82 ± 0.09	1.09 ± 0.02	0.66 ± 0.03
9	S9	*Pseudomonas aeruginosa*	1.028 ± 0.02	1.11 ± 0.03	0.72 ± 0.04
10	S10	*Acinetobacter radioresistens*	0.55 ± 0.03	0.89 ± 0.02	0.28 ± 0.02
11	**S11**	***Pseudomonas aeruginosa***	1.09 ± 0.01	1.12 ± 0.01	0.85 ± 0.03
12	S12	*Bacillus cereus*	1.07 ± 0.02	1.19 ± 0.03	0.91 ± 0.01

*Note:* Data: Mean ± SEM. The experiments were performed in three replicates. Bold entries indicate that *Bacillus cereus* (S6) and *Pseudomonas aeruginosa* (S11) are the most efficient black oil degrading bacteria.

**Table 5:** The chemical combination of black oil (control group) determined by GC-MS analysis. It consists of 25 compounds of aromatic and aliphatic substances.

**No.**	**RT (min)**	**Area %**	**Name**	**Quality**	**CAS number**
1	11.341	0.38	Cyclopentasiloxane, decamethyl-	80	000541-02-6
2	15.362	13.88	Dodecamethylcyclohexasiloxane	93	000540-97-6
3	16.545	0.37	Tetradecane	93	000629-59-4
4	17.894	0.19	n-Eicosane	60	000112-95-8
5	18.61	0.55	Pentadecane	96	000629-62-9
6	19.02	13.08	Dodecamethylpentasiloxane	52	000141-63-9
7	20.561	0.84	Cetane	96	000544-76-3
8	21.536	0.29	Tridecane, n-	90	000629-50-5
9	22.294	6.88	: Cyclooctasiloxane, hexadecamethyl-	59	000000-00-0
10	24.183	2.22	Octadecan	98	000593-45-3
11	25.132	3.17	Cyclononasiloxane, octadecamethyl-	90	109007-87-6
12	25.858	3.06	Nonadecane	99	000629-92-5
13	27.462	5.31	Pentadecane	97	000629-62-9
14	27.68	1.85	Boldenone, di-trimethylsilyl	62	109007-87-6
15	28.998	6.73	n-Heneicosane	98	000629-94-7
16	30.461	8.18	Normal-docosane	99	000629-97-0
17	31.867	6.59	n-Tricosane	99	000638-67-5
18	33.216	6.82	Tetracosane	98	000646-31-1
19	34.518	4.92	n-Heneicosane	98	000629-94-7
20	35.769	4.03	n-Hexacosane	99	000630-01-3
21	36.972	3.28	n-Heptacosane	98	000593-49-7
22	38.13	1.98	Heptacosane, 1-chloro-	98	062016-79-9
23	39.255	1.98	n-Eicosane	95	000112-95-8
24	40.345	1.82	n-Eicosane	96	000112-95-8
25	40.791	1.58	Baccharene	53	036441-74-4

**Table 6:** The residual combination of black oil degraded by *B. cereus* (S6), determined by GC-MS analysis, shows a reduction in black oil components.

**No.**	**RT (min)**	**Area %**	**Name**	**Quality**	**CAS number**
1	11.336	0.58	Cyclopentasiloxane, decamethyl-	83	000541-02-6
2	15.367	37.94	Cyclohexasiloxane, dodecamethyl	94	000540-97-6
3	19.025	30.38	Cyclononasiloxane, octadecamethyl-	46	013450-70-9
4	22.299	16.72	Tetracosamethyl-cyclododecasiloxane	58	018919-94-3
5	25.132	10.29	Cyclononasiloxane, octadecamethyl-	74	109007-87-6
6	30.015	1.71	Tetracosamethyl-cyclododecasiloxane	53	018919-94-3
7	30.45	0.57	Heptadecane	93	000629-78-7
8	31.862	0.58	Nonadecane	96	000629-92-5
9	32.147	0.72	Cardigin	93	109007-87-6
10	33.211	0.51	n-Eicosane	96	000112-95-8

**Table 7:** The residual combination of black oil degraded by *P. aeruginosa* (S11), determined by GC-MS analysis, shows a reduction in black oil components.

**No.**	**RT (min)**	**Area %**	**Name**	**Quality**	**CAS number**
1	11.34	0.85	Cyclopentasiloxane, decamethyl-	91	000541-02-6
2	15.367	37.80	Dodecamethylcyclohexasiloxane	94	000540-97-6
3	19.025	31.24	1,11-Dihydrogendodecamethylhexasiloxane	50	000995-82-4
4	22.299	14.17	6-Aza-5,7,12,14-tetrathiapentacene	64	000000-00-0
5	25.132	6.49	Cyclononasiloxane, octadecamethyl-	49	000556-71-8
6	27.456	0.75	Pentadecane	91	000629-62-9
7	27.674	2.80	Heptasiloxane, hexadecamethyl-	53	019095-24-0
8	28.987	0.77	Eicosane	92	000112-95-8
9	30.02	1.88	6-Aza-5,7,12,14-tetrathiapentacene	47	000000-00-0
10	30.455	0.96	Normal-heptadecane	98	000629-78-7
11	31.862	0.69	Octadecan	98	000593-45-3
12	32.147	0.53	Morphine, bis(trimethylsilyl) ether	74	109007-87-6
13	33.216	0.62	Heneicosane	96	000629-94-7
14	35.758	0.44	Docosane	95	000629-97-0

## Data Availability

The data used to support the findings of this study are available from the corresponding author upon request.
